# A Rare Case of Tension Empyema Unveiling the Diagnostic Pitfalls of Pneumococcal Rapid Urinary Antigen

**DOI:** 10.7759/cureus.84153

**Published:** 2025-05-15

**Authors:** Louay Aldabain, Metri Haddaden, Bazil Tariq, Taha Khalid, Bisher Haddadin

**Affiliations:** 1 Hospital Medicine, MedStar Good Samaritan Hospital, Baltimore, USA; 2 Pulmonary and Critical Care Medicine, University of Oklahoma Health Sciences Center, Oklahoma City, USA; 3 Internal Medicine, George Washington University School of Medicine and Health Sciences, Washington, DC, USA; 4 Medicine, Services Hospital, Services Institute of Medical Sciences, Lahore, PAK; 5 Medicine, Mu'tah University, Mu'tah, JOR

**Keywords:** cross-reaction, empyema, false positive, pneumococcal rapid urinary antigen test, streptococcus viridans, tension empyema

## Abstract

Tension empyema is a rare, life-threatening condition characterized by infected pleural effusion leading to significant respiratory distress, mediastinal shift, and hemodynamic instability. We present a unique case of a 55-year-old male with a history of alcohol use disorder who presented with productive cough, hemoptysis, shortness of breath, and left-sided back pain persisting for over one month. Imaging demonstrated a unilocular fluid collection in the left upper anterior pleural space and a large loculated left-sided hydropneumothorax causing mediastinal shift, raising concern for tension empyema. The patient was successfully managed with thoracentesis, chest tube drainage, intrapleural fibrinolytic and mucolytic therapy, and targeted antibiotic therapy, thus avoiding surgical intervention. Notably, the pneumococcal rapid urinary antigen test was positive, whereas the pleural fluid culture yielded *Streptococcus viridans*. This case underscores the importance of clinical suspicion, rapid diagnosis, appropriate microbial sampling, and timely management of tension empyema. Additionally, it highlights a potential diagnostic pitfall associated with the pneumococcal rapid urinary antigen test, possibly due to molecular mimicry between the two organisms, a phenomenon not previously reported in the literature.

## Introduction

Parapneumonic effusion refers to fluid accumulation within the pleural cavity secondary to pneumonia [1]. When this fluid becomes infected, it evolves into empyema, leading to pus formation in the pleural cavity [1]. *Streptococcus* is responsible for approximately 50% of community-acquired pleural fluid infections, with the most common being *Streptococcus intermedius* and *Streptococcus pneumoniae*, while anaerobic bacteria are found in 20% of the cases [2].

A rare yet severe complication of empyema is a phenomenon known as tension empyema. The latter results from increased pleural pressure leading to severe respiratory distress, mediastinal shift, or hemodynamic compromise due to decreased venous return [3]. The incidence of empyema as a complication of pneumonia in hospitalized patients is approximately 5.98 per 100,000 cases per year in the United States [4]. However, our literature review identified only 13 previously reported cases of tension empyema [5-17]. Here, we report a unique case of tension empyema in a 55-year-old male with alcohol use disorder, whose diagnostic evaluation intriguingly revealed a positive urinary antigen test for *Streptococcus pneumoniae* despite pleural fluid cultures yielding *Streptococcus viridans*, raising concern for molecular mimicry between these organisms.

This case was presented at the CHEST conference as a poster on October 10, 2023, under the title “Streptococcus viridans tension empyema in a patient with a false-positive pneumococcal urinary antigen test.”

## Case presentation

**Figure 1:**
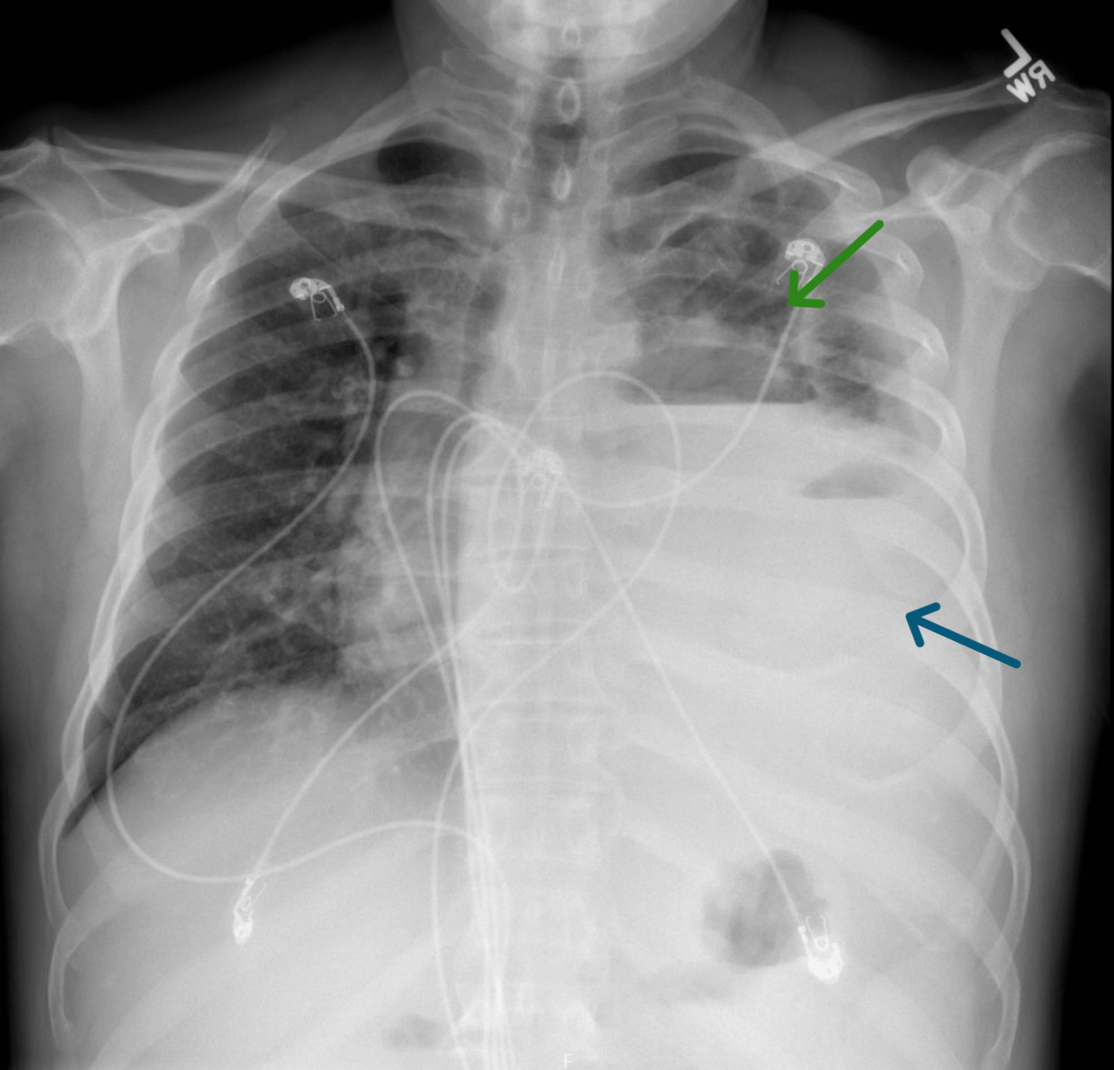
Chest X-ray showing a large left loculated hydropneumothorax with mediastinal shift to the right (blue arrow) and a questionable 5 cm left supra-hilar mass (green arrow).

**Figure 2:**
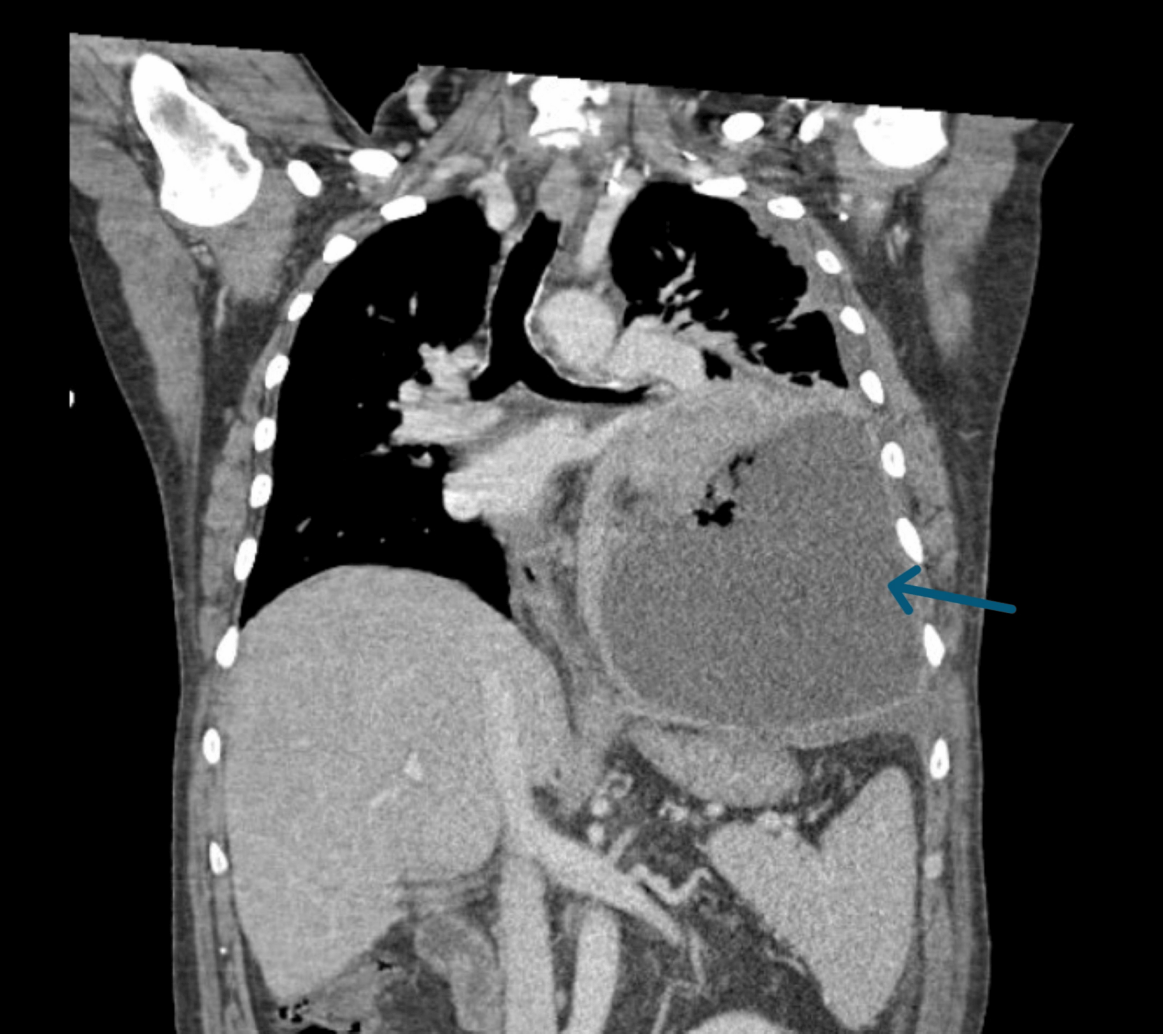
CT of the chest, coronal view, demonstrating a large left-sided pleural effusion with a rightward mediastinal shift (blue arrow).

**Figure 3:**
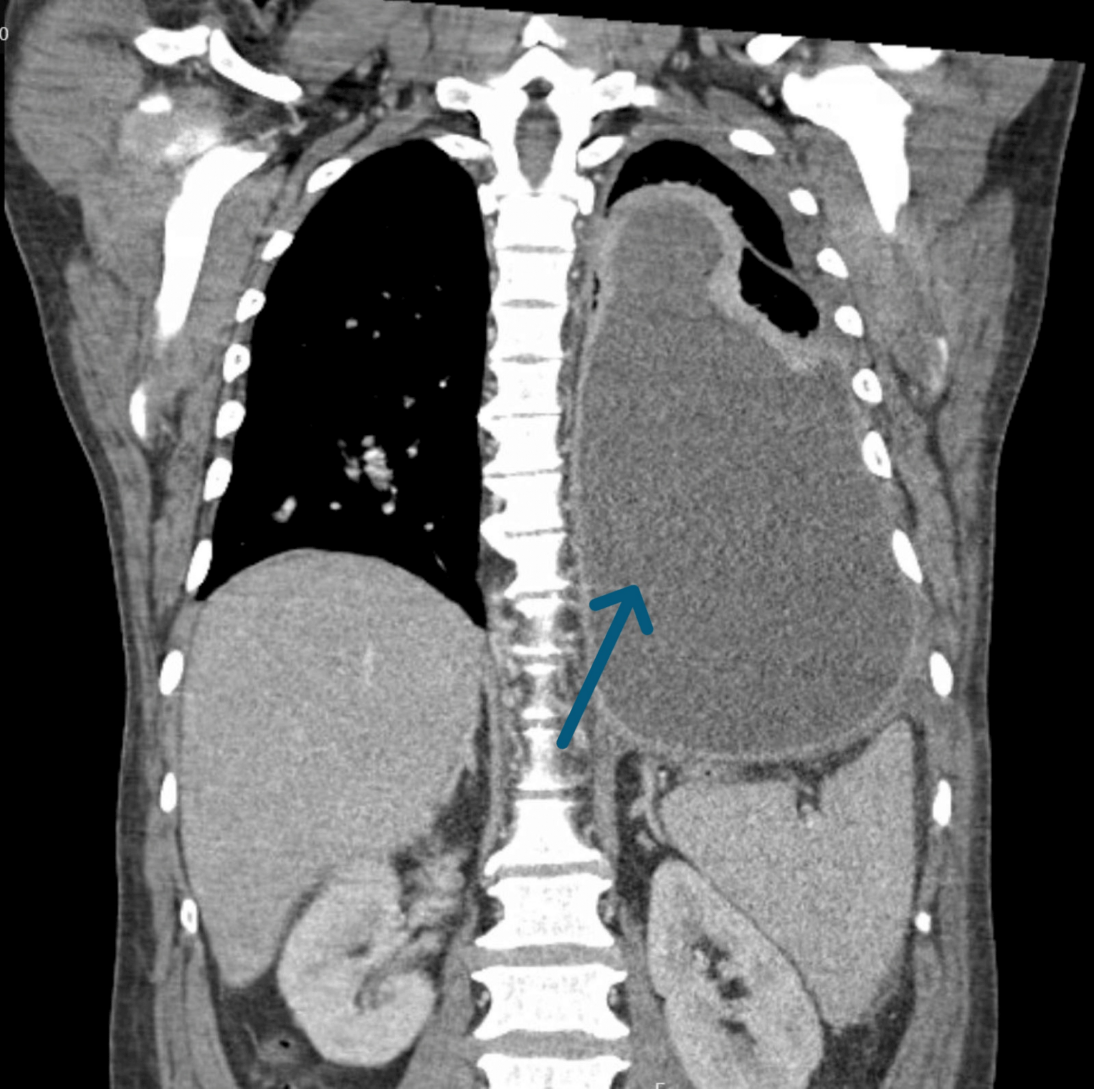
CT of the chest, another coronal view, demonstrating the large left-sided loculated pleural effusion (blue arrow).

**Figure 4:**
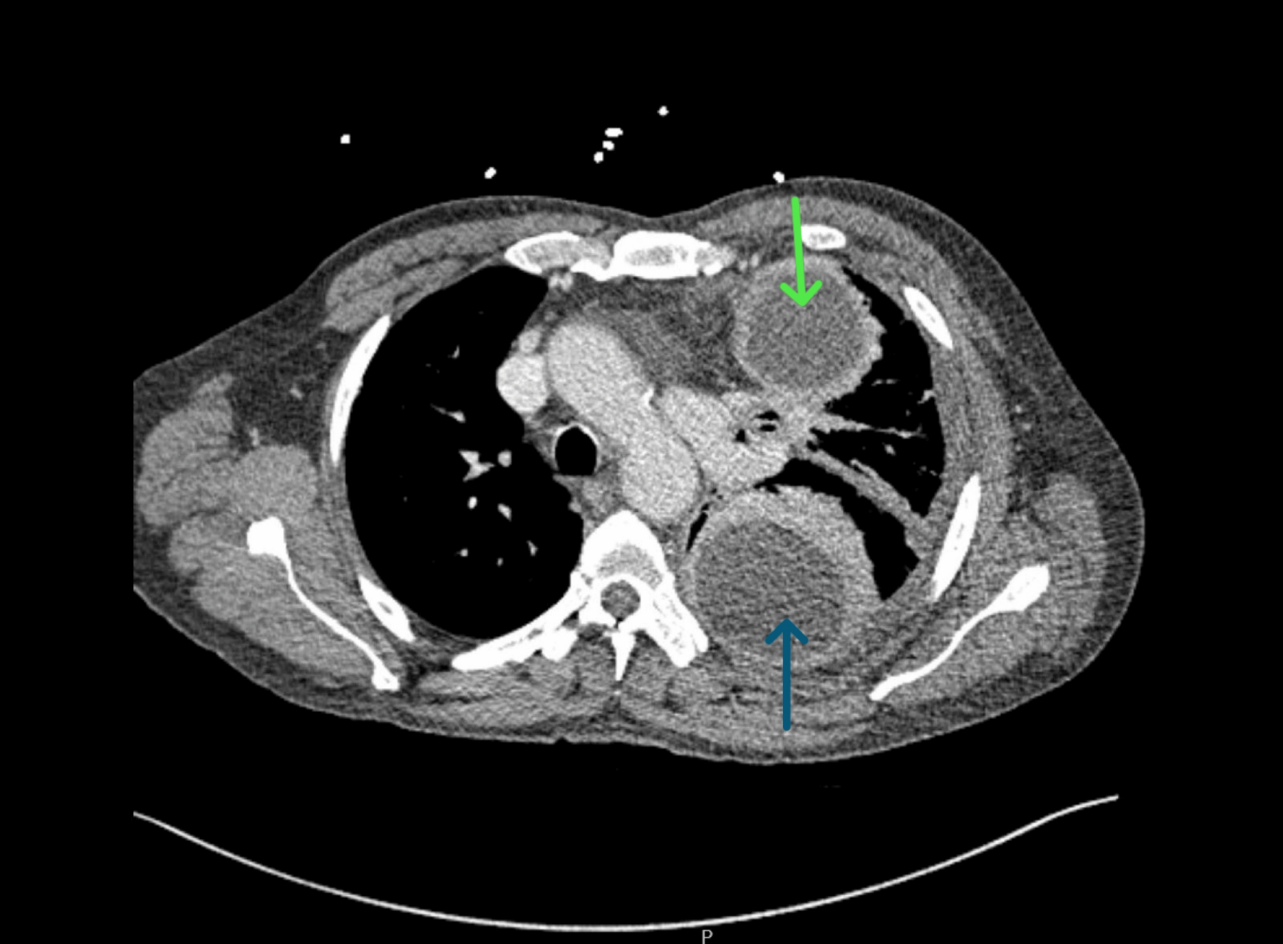
CT scan, axial view, demonstrating a large left-sided loculated pleural effusion with a rightward mediastinal shift (blue arrow) and a small discrete fluid collection in the left upper anterior pleural space.

A 55-year-old male patient with alcohol use disorder presented to the emergency department, complaining of productive cough, hemoptysis, shortness of breath, and upper left back pain for over a month, but had continued to disregard them. He eventually sought care at the hospital after developing diarrhea and reduced oral intake along with the previously mentioned symptoms. The patient denied any history of smoking. His drinking habits involved multiple episodes of binge drinking, leading to complete loss of consciousness, with the last episode reported a month before his presentation. In the emergency department, the patient was found to be afebrile, tachycardic with a heart rate of 110 beats/minute, blood pressure of 141/76 mmHg, respiratory rate of 22 breaths/minute, and oxygen saturation of 93% on room air. On examination, he was alert and oriented, with dry oral mucosa, and absent breath sounds on the left side of the chest. The rest of the examination was unremarkable. His laboratory findings are presented in Table 1. The *Streptococcus pneumoniae* urinary rapid antigen test was positive. Chest X-ray (Figure 1) showed a large left loculated hydropneumothorax with a mediastinal shift to the right and a questionable 5 cm left supra-hilar mass. Subsequently, CT of the chest, abdomen, and pelvis with intravenous (IV) contrast (Figures 2-4) was performed, demonstrating a large loculated left-sided pleural effusion with air-fluid level compressing the left bronchi, rightward mediastinal shift, thick enhancing ring, a small discrete fluid collection in the left upper anterior pleural space, right upper lobe ground-glass opacities, and a nodular liver contour, suggesting cirrhosis.

**Table 1:** Laboratory results.

Laboratory test	Laboratory value	Reference range
Sodium	131 mmol/L	136–145 mmol/L
Aspartate transaminase	117 U/L	0–33 U/L
Alanine transaminase	72 U/L	10–29 U/L
Alkaline phosphatase	217 U/L	46–116 U/L
White blood cell count	15.35 k/µL	4–10.8 k/µL

**Figure 5:**
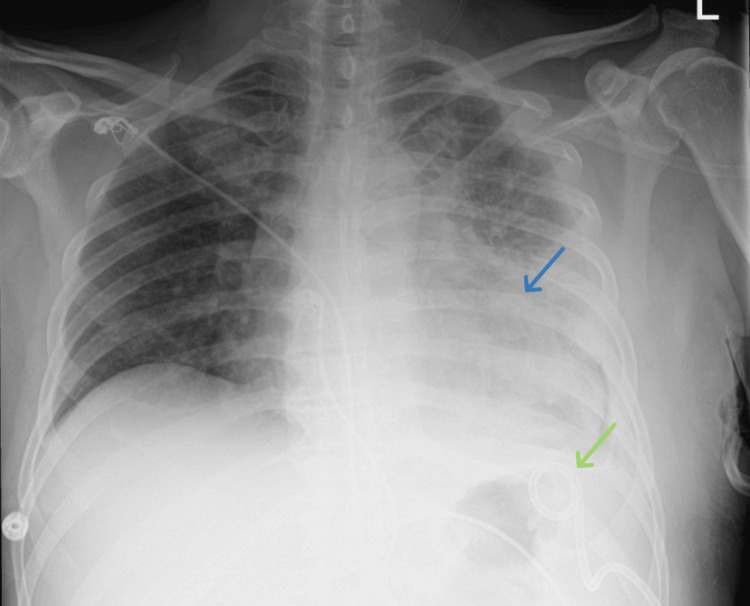
Portable chest X-ray after chest tube insertion (green arrow) showing significant interval drainage of the left hemithoracic hydropneumothorax (blue arrow) and mild residual retrocardiac left lower lobe airspace opacity.

The patient underwent aspiration of the unilocular collection in the left upper anterior pleural space, yielding 70 mL of purulent fluid, as well as a 14-French pigtail chest tube insertion, which yielded 2.2 L of turbid purulent fluid. The details of pleural fluid analysis are provided in Table 2. The post-procedural chest X-ray confirmed chest tube placement and the resultant improvement of the left hemithoracic hydropneumothorax (Figure 5).

**Table 2:** Pleural fluid analysis laboratory results.

Pleural fluid analysis	Laboratory result
Fluid appearance	Turbid
Fluid color	Purulent
pH	5.8
Protein	Less than 2 g/dL
Albumin	Less than 1
Lactate dehydrogenase	3,957 U/L
Glucose	7 mg/dL
White blood cell count	142,414/mm^3^
Red blood cell count	224,000/mm^3^
Pleural fluid culture	Positive for *Streptococcus viridans*

He reported significant symptomatic relief after pleural fluid removal. The chest tube was placed on suction at -20 cm H_2_O and was maintained with daily flushes. Intrapleural therapy of combined tissue plasminogen activator (tPA) and human recombinant deoxyribonuclease (DNase) was administered. The patient initially received piperacillin/tazobactam, and then antibiotics were narrowed down to ampicillin/sulbactam. A repeat CT scan on day five showed near resolution of the ground-glass infiltrate in the anterior right upper lobe, a marked interval decrease in the complex loculated left hydropneumothorax, and a reduction in the size of the anterior left upper lobe loculated empyema. However, a moderate left lower lobe infiltrate and a small residual left hydropneumothorax were still seen. The thoracic surgery team also evaluated the patient and recommended continuing medical management. Surgical intervention was deferred as the patient responded to fluid drainage and antibiotics. The chest tube was removed on day nine, and the patient was discharged on amoxicillin/clavulanic acid for seven days with outpatient follow-up with thoracic surgery.

## Discussion

Tension empyema is a rare but life-threatening condition. This case highlights the critical importance of promptly recognizing risk factors, identifying clinical manifestations, initiating rapid diagnostic evaluation, and timely intervention to ensure favorable patient outcomes and prevent fatal complications.

Multiple factors have been identified as independent predictors for the development of complicated parapneumonic effusion or empyema. These include low serum albumin (<30 g/L), C-reactive protein >100 mg/L, platelet count >400 × 10^9^/L, serum sodium <130 mmol/L, IV drug use, and chronic alcohol abuse [18]. In our case, the patient’s history of alcohol use disorder was a significant risk factor, and his clinical presentation, including a productive cough, hemoptysis, back pain, and respiratory distress with absent breath sounds in the left hemithorax, raised a high index of suspicion for this life-threatening condition. Prompt diagnostic evaluation revealed a unilocular fluid collection in the left upper anterior pleural space, a large loculated pleural effusion, and mediastinal shift on chest CT. The patient was swiftly managed with broad-spectrum antibiotics, aspiration of the loculated left upper anterior pleural collection, thoracentesis, and tube thoracostomy, resulting in immediate symptomatic improvement.

Management of empyema includes thoracentesis with pleural drain placement, antibiotics (parenteral second or third-generation cephalosporins with metronidazole, parenteral aminopenicillin with β-lactamase inhibitor) [1]. Final antibiotic therapy can be tailored based on culture results [1]. However, continuing anaerobic coverage empirically can be considered even when anaerobic cultures are negative [1]. Due to the lack of comparative trials, empyema is usually treated within two to six weeks of antibacterial therapy [1]. The duration of antibiotic therapy should be individualized based on the organism, adequacy of source control, and the patient’s clinical response to treatment, with a minimum of two weeks from the time of drainage [1]. After tube thoracostomy, it is recommended to perform routine drain flushing of the tube and repeat a close CT follow-up to confirm adequacy of drainage [1]. Evidence supports the use of the combination of intrapleural tPA 5 mg and DNase 10 mg for three days to improve fluid drainage and reduce the frequency of surgical intervention and the length of hospital stay [19]. Thoracic surgery evaluation is essential in all patients with empyema requiring chest tube insertion. Surgical management should be highly considered in patients with ongoing sepsis along with persistent pleural collection despite drainage and antibiotic administration [1]. The choice of procedure must factor in the patient’s age, comorbidities, availability of equipment, and the surgeon’s preference [1]. Recently, video-assisted thoracoscopic surgery has been considered as a first-line therapy, but open thoracic drainage or thoracotomy and decortication can be considered alternative techniques [1].

Interestingly, our patient had a positive urine *Streptococcus pneumoniae* antigen, but his pleural cultures grew *Streptococcus viridans*. Pneumococcal rapid urinary antigen tests have a sensitivity of 74% and a specificity of 94% for *Streptococcus pneumoniae*, making them a useful diagnostic tool in cases where invasive testing may be risky or not feasible [20]. It is important to note that possible cross-reactions with some α-hemolytic oral streptococci such as *Streptococcus mitis* and *Streptococcus oralis* can occur, and false-positive pneumococcal rapid urinary antigen tests have been reported in infections caused by *Streptococcus intermedius* and *Streptococcus constellatus* [20-22]. However, we did not find any reported cases of such cross-reactivity with *Streptococcus viridans*, which makes this case unique.

## Conclusions

This case emphasizes the importance of clinical suspicion, rapid diagnosis, appropriate microbial sampling, and timely intervention in managing tension empyema to minimize morbidity and prevent mortality. It also highlights potential diagnostic pitfalls associated with pneumococcal rapid urinary antigen tests, making it crucial to obtain appropriate cultures to guide targeted antibiotic therapy in such cases. Further research is needed to prove molecular mimicry between *Streptococcus pneumoniae* and *Streptococcus viridans*.
